# Impact of Thermal Properties on Crystalline Structure, Polymorphism and Morphology of Polymer Matrices in Composites

**DOI:** 10.3390/ma14092136

**Published:** 2021-04-22

**Authors:** Maria Raimo

**Affiliations:** Institute for Polymers, Composites and Biomaterials, National Research Council, Via Campi Flegrei 34, 80078 Pozzuoli, Italy; maria.raimo@cnr.it

**Keywords:** thermal effects, polymer composite, structure, morphology, solidification, thermal conductivity, columnar growth, nucleation

## Abstract

Morphological analysis at different levels is fundamental to understand properties of materials, as these latter are dictated not only by the chemical composition but also by the shape. Solid structures arise from a balance between thermodynamic and kinetic factors, which, especially for polymer composites, depend also on interactions amongst components. In particular, morphology is strongly affected by the heat transfer pattern during crystallization and by the difference in thermal behavior between polymer matrix and filler. Polymers show a spherulitic structure, arising from the start of crystallization in several points of the liquid phase. Within a general rounded shape, spherulites show variability in growth patterns, morphology, and geometry of boundaries. The appearance and the number of spherulites, as well as their growth mechanism, may vary not only in dependence of the chemical composition and the crystalline structures but also, for a same polymer, in consequence of experimental conditions and incorporation of fillers. This article reviews the crystallization process of polymer matrices in the framework of crystal growth and heat transport theories, and explains microstructural differences between composites and neat matrices on the basis of the differences in thermal capacity and conductivity between polymers and additives.

## 1. Introduction

**Figure 1:**
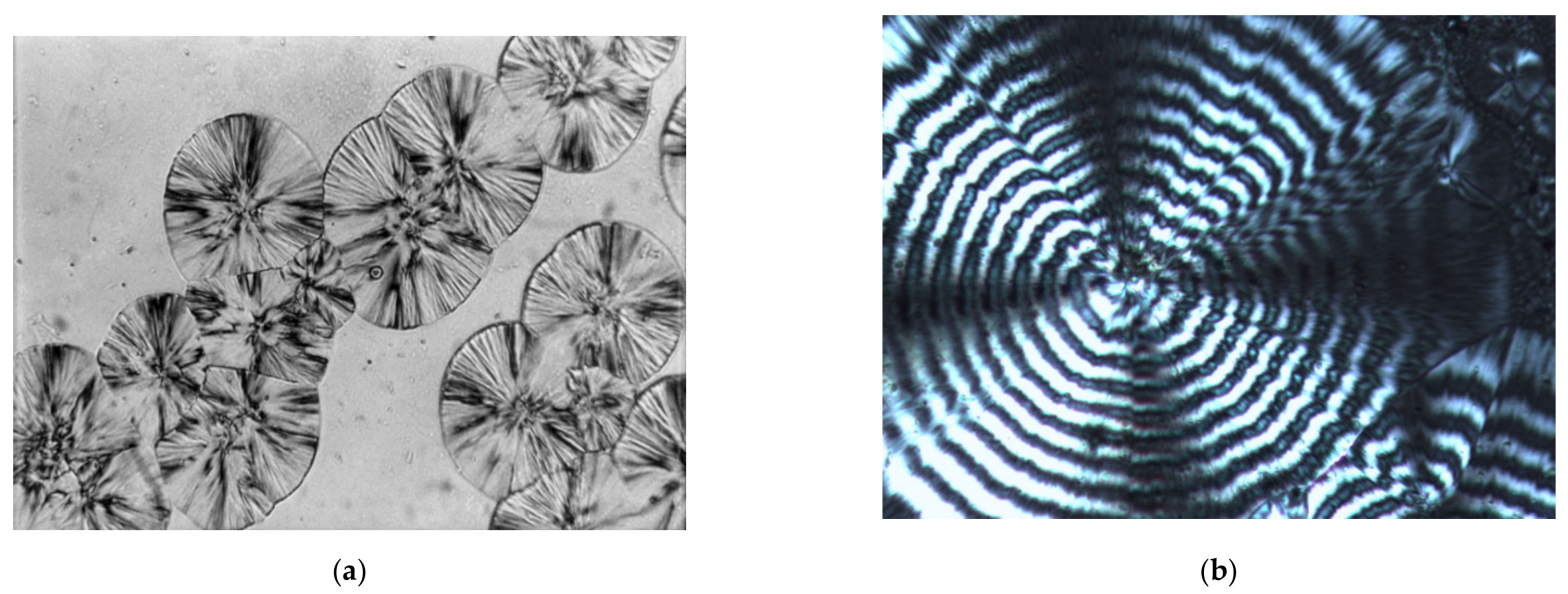
(**a**) Optical micrograph taken during crystallization of poly(ε-caprolactone), parallel Nicols, and (**b**) micrograph of a spherulite of poly(3-d-hydroxybutyrate), crossed Nicols.

The solid state has been subjected to morphological investigations of long-held interest, as the shape plays an important role in determining the mechanical resistance of solids and almost all industrial processes involve solidification. Morphology of crystalline substances can be analyzed at different length scales, ranging from macroscopic size to molecular and atomic positions and distances. At microscopic scale, polymers show a spherulitic structure, arising from the start of crystallization (i.e., nucleation) in several points of the liquid phase. A spherulite is a polycrystalline entity growing with a rounded shape up to impingement with surrounding spherulites [1]. For instance, in Figure 1, spherulites of poly(ε-caprolactone) (PCL) and poly(3-d-hydroxybutyrate) (PHB), respectively, are shown.

**Figure 2:**
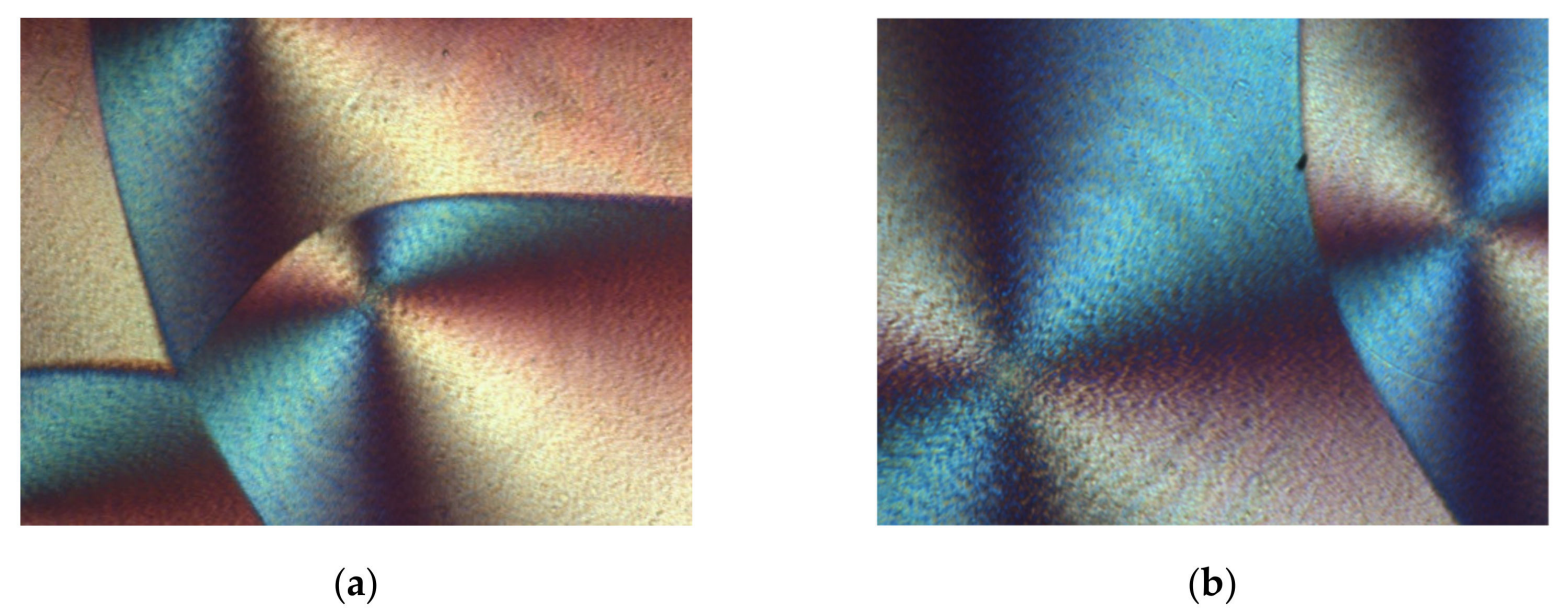
(**a**) Micrograph of four impinged poly(3-d-hydroxybutyrate) (PHB) spherulites, separated by a line (left bottom) and branches of hyperbola according to their nucleation times and (**b**) micrograph showing two PHB spherulites nucleated at slightly different times.

Coalescence leads to a tessellated structure where the original spherulites are separated from one another by interfaces, whose analytical shape depends on the relative overall growth rates of adjacent spherulites. Figure 2 shows hyperbola-shaped and linear interfaces between couples of adjacent PHB spherulites growing at the same linear rate but nucleated, respectively, at different times or simultaneously.

To discuss some aspects of crystallization, the crystalline structure has to be considered [2,3,4], since the prediction of the type of phase and morphology originated by a substance cannot be made without taking into account parameters connected to local crystal order and symmetry. Such parameters affect the properties not only of crystals, but also of crystal aggregates as spherulites. Non-symmetric crystalline lattice, for instance, causes the appearance of four birefringent sectors in spherulites that, because of radial arrangement of elongated crystals, are separated by a distinctive dark cross characteristic of extinction. Although mainly determined by the molecular and crystalline structures, the physical properties of a material depend considerably also on the microstructure and orientation generated by solidification; the microstructure and properties of composite materials are affected, in turn, by the shape, orientation, dispersion, and thermal properties of fillers.

The relative position of atoms within an ideal solid is defined by a crystalline entity (the unit cell) whose translation in the three spatial dimensions reproduces the whole solid structure (the space lattice). Therefore, the description of the crystalline structure of a polymer is based on the inherent admission that a regular order along three spatial directions is present. As underlined by Natta and Corradini [5], the crystal structure of linear polymers follows the same principles derived for proteins by Pauling [6]. Therefore, each single polymer chain has to have a periodic structure not only constitutionally and configurationally but also from a conformational point of view. In other words, polymers are able to crystallize only if the need of geometric equivalence, relatively to a chain axis, of the structural units may be achieved. This entails that the macromolecular axis (coinciding, for helical conformations, with the helical axis) is parallel to one of the crystallographic axes [5].

**Figure 3:**
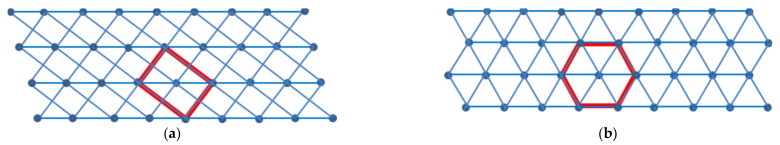
(**a**) Packing of polyoxymethylene (POM) chains in the orthorhombic lattice and (**b**) packing of POM chains in the hexagonal or rhombohedral lattice. After references [7,8,9].

Often a substance can crystallize in more than one solid phase, originating polymorphs with different crystalline structures and, hence, different physical properties. Polymorphs may be related by a monotropic or an enantiotropic relationship. In the former case, one polymorph is metastable, whereas in the latter case, each polymorph has its own stability range and, at least theoretically, a definite transition temperature between the two forms exists. In Figure 3, the two crystalline structures of polyoxymethylene (POM), as schematic projections on a plane perpendicular to the chain axis, are shown.

POM helices are orthogonal to the plane of the figure and parallel to the crystallographic *c* axis. The orthorhombic unit cell contains, as a whole, two polymer chains, since four of the five pieces of helices belong to the unit cell for 1/4. The pieces of helices in the orthorhombic crystal contain 2 monomeric units CH_2_-O- for each complete turn, which defines the size of the cell along the vertical *c* axis. The hexagonal form (*a* = *b* ≠ *c*, α = β = 90°, γ = 120°) is the low-density crystalline phase of POM and represents the common form of the solid polymer, but chains can be also arranged in an orthorhombic crystal lattice (*a* ≠ *b* ≠ *c*, α = β = γ = 90°). The wide-angle X-ray diffraction curve of non-oriented hexagonal POM shows four peaks at 2θ equal to 22.9°, 34.6°, 48.4°, and 54.1° corresponding to lattice planes with Miller indexes (100), (105), (115), and (205). The fiber pattern of orthorhombic POM shows instead peaks at angles of 21.9°, 37.7°, and 23.2°, indexed as (110), (200), and (020) reflections, respectively. The choice of a hexagonal unit cell for the common phase of POM has the advantages to reproduce the external crystal geometry (i.e., the crystal habit) and the whole properties of the macroscopic solid. However, smaller primitive or elemental cells can be used to reproduce easily the whole lattice. The elemental cell of hexagonal POM, indeed, may be considered of rhombohedral type. Each chain in a primitive rhombohedral cell belongs to four adjacent cells, so that each cell can be considered occupied by the volume of a single polymer chain. Moreover, each chain is shaped as a 9/5 helix (the repeating unit of the helix is made up of 9 chemical units coiled into 5 turns). The orthorhombic POM cell, which corresponds to a rectangle in the *ab* plane, contains instead two 2/1 helices [7] (a 2/1 helix can be considered as a 10/5 helix and, therefore, is shorter and wider than a 9/5 helix with the same number of constitutional units CH_2_O). It is worth noting that the orthorhombic phase is the high-density crystalline form of POM because of the high compactness of 2/1 helices along the *c* axis. According to the density rule [10], the less dense hexagonal form is the less stable phase at low temperature and the most stable phase at high temperatures. By heating, indeed, the high-density orthorhombic form is found to undergo an endothermal transformation (at about 70 °C) to the less dense hexagonal form, a further evidence of an enantiotropic relationship between the two POM crystalline forms [10]. This endothermal phase transformation indicates that the orthorhombic POM has higher stability below 70 °C, whereas at higher temperatures, the thermodynamically stable phase is hexagonal. Differently from monotropic polymorphs, the enantiotropic POM modifications have two temperature ranges of stability and, in principle, under atmospheric pressure, a reversible transition from one form to the other at a fixed temperature should be observed, because of the stability inversion. However, thermodynamically permitted processes may be hindered by kinetic reasons, so that chemico-physical procedures do not necessarily lead to the most stable products. For instance, when solid POM is obtained at temperatures above the transition temperature, it is likely found in the hexagonal phase and, because of the very slow tendency to transform in the orthorhombic phase, it remains in the same crystal structure also by subsequent cooling to ambient temperature. Orthorhombic POM, however, can be directly obtained at temperature below 70 °C: under this condition, it is not only the most stable product but it is also formed faster than the less stable hexagonal phase. As shown in Figure 3, the orthorhombic-hexagonal conversion needs a slight expansion along the *b* axis and a contraction along the a axis of the orthorhombic phase. It is worth underlining that in the orthorhombic phase, the distance between the central helix and each of the other four helices of the cell is 4.51 Å, whereas the distance between the central helices of two adjacent orthorhombic cells is 4.77 Å. Comparing the two crystal structures, the small expansion of the primitive orthorhombic cell along the *b* axis and the contraction along an a axis, needed to transform the orthorhombic phase into the hexagonal phase, become evident. The distortion needed for the orthorhombic-hexagonal transformation leads to the conversion of the isosceles triangles, formed by any three adjacent helices, in equilateral triangles. The inverse hexagonal-orthorhombic phase transformation, although possible in principle even at ordinary pressure by cooling the hexagonal form at a temperature below 70 °C, has not been observed, likely because the needed structural changes are hindered or extremely slow at low temperatures and pressure. However, at least in theory, it might be possible to obtain crystals of orthorhombic POM from the rhombohedral phase, by recrystallization at temperatures lower than 70 °C after dissolution of the latter phase in an appropriate solvent. Crystallization from melt, instead, favors the hexagonal phase, since this latter is formed rapidly during cooling, before temperatures lower than 70 °C have been reached.

Kinetics and thermodynamics of liquid–solid transitions are very sensitive to small variations of experimental conditions, and this is even more evident for polymers that usually show constitutional, configurational, and conformational variability. The presence of metallic and ceramic particles or fibers alters heat exchange within polymer matrices, producing thermal effects during crystallization and the formation of distinctive morphology. Indeed, composites may show not only polymorphic crystalline structures not easily found in neat matrices but also morphology and thermal behavior different from those observed in the neat matrix crystallized under the same conditions. Hereinafter, polymer crystallization and morphology will be examined in order to compare the microstructure of composite materials to that of the neat matrices. This article reviews the crystallization process of polymer matrices in the framework of crystal growth and heat transport theories and explains microstructural differences between composites and neat matrices on the basis of the differences in thermal capacity and conductivity between polymers and additives. An intuitive approach, although rigorously based on physico-mathematical concepts, has been undertaken for the sake of simplicity. Previous reviews cover several other specific aspects of polymer crystallization. Here, instead an overall view, including not only basic theories and models but also fundamental issues often forgotten or neglected in polymer science, has been offered in order to stimulate deeper investigation of thermal effects of great practical potentiality and make easier recognition and explanation of thermal phenomena and morphology occurring in polymers and their composites. Indeed, a general view of solidification favors the right connections amongst morphology and thermal, structural, and stereochemical properties of macromolecular chains, avoiding the risk of incorrect data interpretation. In order to encompass a wide range of readers from different fields, the theoretical background includes basic concepts of stereochemistry and the general theory of crystallization, which are essential for the comprehension of distinctive properties of polymorphs and of the strictly interconnected kinetic and thermodynamic aspects of solidification. Without a deep knowledge of all the factors affecting the crystallization of neat polymer matrices, it is, indeed, impossible to clearly link the crystallization changes of the matrix to thermal effects due to fillers. According to their expertise, readers may decide to skip some side issues aiming the attention towards the final paragraphs.

## 2. Theoretical Background

### 2.1. Enantiomorphism and Polymorphism

**Scheme 1:**
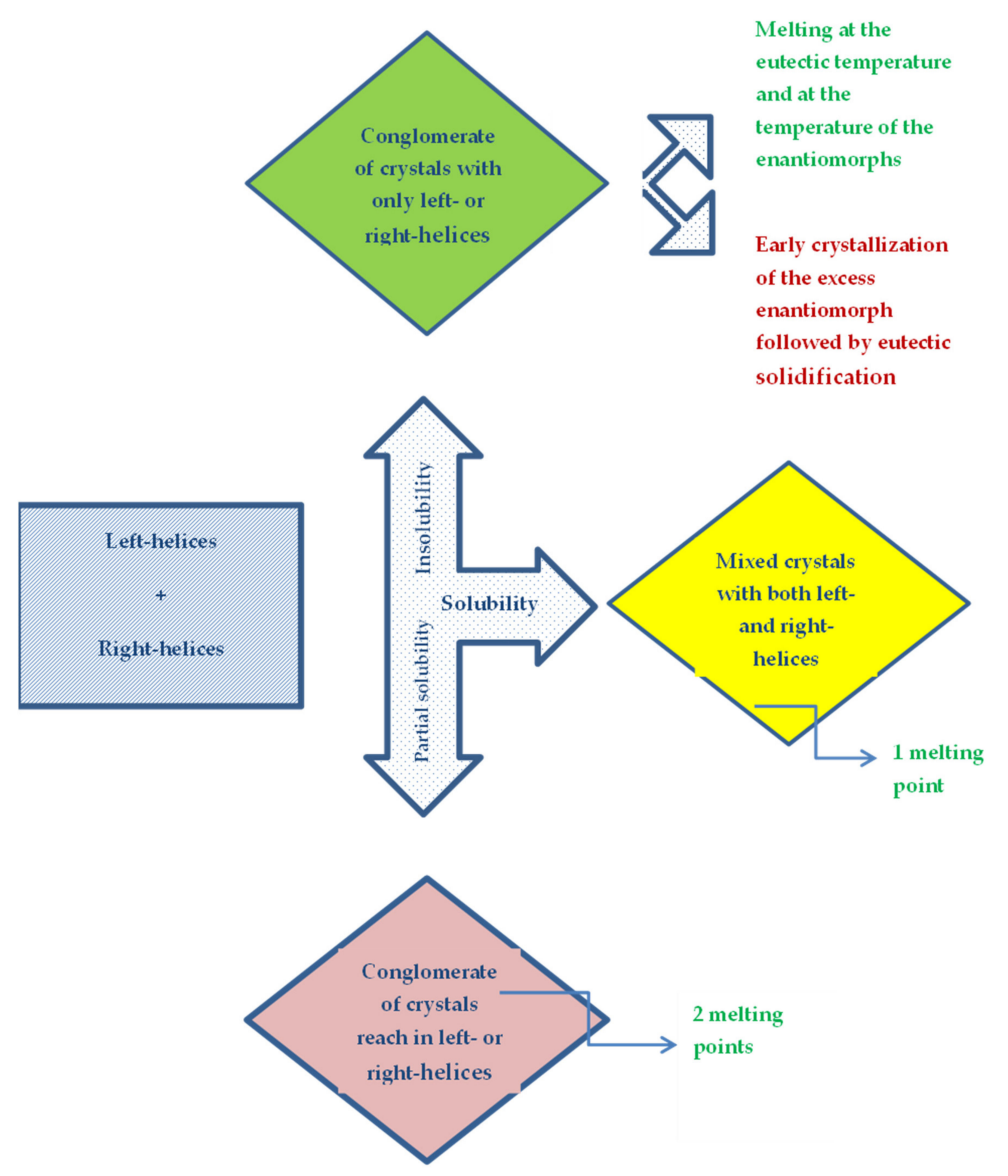
Crystallization and thermal behavior of crystals containing enantiomeric helices.

A basic concept, related not only to the presence of asymmetric centers but also to chains shaped into a helix, is chirality. A molecule shaped as a helix is inherently chiral, since right-handed and left-handed helices are not superimposable mirror images. Right-handed and left-handed helices with opposite chirality constitute enantiomorphs, which are able to rotate the plane of linearly polarized light (a characteristic referred to as optical activity or circular birefringence) of the same amount but in opposite directions. The possibility of conversion of one enantiomorph helix into the other through conformational changes depends on the type of bonds between atoms along the chain and on the presence of lateral groups. The conversion is usually possible when single bonds follow one another and the molecules do not present large lateral groups. Therefore, flexible conformational enantiomorphs in the liquid state or solution interconvert continuously one into another at ordinary temperatures and cannot be separated. In contrast, enantiomorphic helices of rigid molecules, as hexahelicene (an aromatic compound, with molecular formula C_26_H_16_, formed by six ortho-condensed benzene rings), and helices of flexible macromolecules in the solid state cannot be interconverted [11], unless covalent bonds in a helix are disrupted and reassembled according to the opposite helical orientation. Carazzolo [8,9] stated that solid POM, differently from isotactic polypropylene (iPP), should be made up of crystals consisting of all chains with the same handedness (i.e., isomorphous chains), since there is not possibility of alternation or substitution between right-handed and left-handed helices in the same crystalline lattice [9]. Right-handed and left-handed helices of non-chiral macromolecules, or of highly stereoregular syndiotactic polymers, are isoenergetic and, therefore, should be equally probable [12,13,14]. Therefore, when isoenergetic right-handed and left-handed helices cannot be arranged in the same crystalline lattice, crystallization should lead to a conglomeration (i.e., a mixture) formed by 50% of each chiral crystal. If crystals are randomly oriented, this mixture is not able to produce a rotation of the plane of polarized light, since 50% of crystals deviate the plane clockwise and the remaining crystals produce an anticlockwise rotation of the same angle. However, when chiral centers, both in the macromolecules themselves or in impurities, are present, a handedness prevails and chiroptical properties arise. Information on the chirality of crystals formed by helical chains can often be drawn by simple melting point measurements. Indeed, a conglomeration of two chiral crystals shows a melting temperature lower than that of optically pure enantiomorphs and a broader temperature range of fusion. Moreover, if a 50% mixture of the two enantiomorphs corresponds to a eutectic, mixtures with various proportions of the two enantiomorphs will began to melt at the eutectic temperature and, by further heating, fusion will be completed at the melting temperature of the excess enantiomorph. Vice versa, by cooling a mixture of two enantiomers, the solidification of the enantiomorph in exceedance occurs early, until the composition of the liquid phase equals that of the eutectic point so that both the enantiomorphs crystallize separately. The crystallization and thermal behavior of enantiomeric helices is described in Scheme 1.

Poly(3-d-hydroxybutyrate) is an optically active and biodegradable polymer with a perfect isotactic structure, found as energy reserve in some bacteria. In its common crystal structure, PHB chains form 2/1 left-handed helices packed in an orthorhombic cell. Since natural PHB contains asymmetric centers with only the R configuration, PHB helices with the different handedness are not energetically equivalent. PHB including asymmetric centers with only one type of R or S configuration can be obtained synthetically. Racemic mixtures of two equal amounts of PHB with opposite configurations do not show optical activity in solution, since the two configurational enantiomers rotate the plane of polarization of the same angle in opposite directions. Crystallization of a PHB racemic mixture can theoretically lead to a conglomerate of separate R and S crystals, to a racemic compound or to a solid solution. Since R-PHB crystallizes according to a left-handed helix, whereas S-PHB likely forms right-handed helices in the solid state, and moreover, as these two types of helix are not enantiomorphs from a conformational point of view, conglomerates, racemic compounds, and solid solutions are all potentially endowed of optical activity which, therefore, cannot be exploited as a distinctive property of a specific crystallization product. Differently from conglomerations and compounds, a solid solution does not have a melting point significantly different from that of the two separated R and S crystals. A racemic compound, instead, may show a melting point lower or higher than that of the R and S crystals, depending on the force of the interaction between chains with opposite configurations, relatively to the force of the interaction between chains with the same configuration. For instance, blends of poly(l-lactic acid) and poly(d-lactic acid), as well as block copolymers of l and d lactic acid, have been found to form a racemic compound referred to as stereocomplex, with a melting point of 50–60 °C higher than that of homoenantiomers [15]. In this stereocomplex, stronger hydrogen bonds amongst the chains entail a higher density of the solid phase relatively to the enantiomers. When a racemic compound is formed, the phase diagram shows two eutectic points, each formed by the R-enantiomer or S-enantiomer and the same compound. Hypothetical crystals consisting of a PHB racemic compound should include the same amount of R and S chains and, in absence of helical conformations or in presence of helices without preferential handedness, should be optically inactive. However, if each enantiomer chain of a racemic compound is helicoidally twisted according to a specific handedness, so that the contributions of the helices of the two configurational isomers to the optical activity do have not equal intensity and opposite signs, the compound will show optical activity. Analogously, the PHB conglomerate formed by 50% of each enantiomorph with a unique handedness, should show circular birefringence, since the chiroptical sign and value for the blend is determined by the twisting direction of the diastereoisomer helices. It is worth noting that, differently from the melting of a two phases conglomerate, the melting of a racemic compound is incongruent, because it leads to a liquid blend instead that to a liquid compound. All the considerations on the phase diagrams of polymers showing two configurational enantiomorphs are also valid for constitutional polymorphs. For instance, PHB forms a compound with poly(3-hydroxyvalerate) (PHV) and, indeed, PHB-PHV copolymers show a lower melting temperature and crystallinity level [16]. Zhu et al. also studied the melting behavior of a poly(hydroxybutyrate-co-hydroxyvalerate) (PHBV) having 36.2 mol% of hydroxyvalerate (HV) units by in situ synchrotron X-ray analyses [17]. The authors concluded that the low-temperature peak is due to melting of the thinner crystals with lower density because of the HV inclusion in the orthorhombic PHB cells, whereas the high-temperature peak is due to the fusion of the thicker PHB crystals with a small cell excluding HV units, which are confined in the amorphous phase.

Yokouchi et al. reported the structure factor for natural PHB calculated from the intensity of each set of X-ray reflections (the so-called “observed” structure factor, which is defined as the Fourier transform of the electronic density), in comparison with the structure factors calculated from the electronic density of orthorhombic PHB, assumed to have antiparallel two-fold conformations with internal rotation angles of −50, −45, 180, and 149 for left-handed helices and 50, 45, −180, and −149 for right-handed helices [18]. These authors found a good agreement between the calculated and observed structure factors for all the reflections and, on the basis of the similarity of the X-ray diffractions of natural PHB and the racemic mixture of synthetic PHB, assumed that the conglomerate of isotactic PHB consists of two kinds of crystallites: one composed only of helices of the R chain segments and the other composed of helices of the S chain segments.

It is worth noting that, because of the possible helical conformation, chiroptical properties of isotactic chiral polymers, both in solution and in the solid state, do not show a linear dependence from optical activity of the monomers [19]. The prediction of the effect of nanohelices on the optical properties of solids is very difficult since it is necessary to account for their relative amount, orientation, and chirality. For instance, according to Gibbs [20], a racemic solid formed by right-handed helices orthogonal to left-handed helices would rotate the polarization plane of a beam of light entering parallel to one of the two enantiomeric kinds of helix, thus showing optical activity. On the contrary, a beam normal to the plane containing two orthogonal helices with opposite handedness will not produce optical activity. The exact predictions of Gibbs have not attracted the right attention since macromolecular chains have been mainly considered to be always parallel in solids. However, the possibility of a normal arrangement of helices, as that occurring in ɣ isotactic polypropylene [21], exists and cannot be excluded on the basis of the usual assumption of chain-axis parallelism. On the other hand, a solid solution 50/50 weight by weight of R and S crystals, as well as a racemic conglomerate or compound of R and S crystals, will lose possible optical activity in the liquid phase or in solution, where polymer chains adopt a random coil conformation, and moreover, possible left-handed and right-handed segments of helices may interconvert. Fortunately, the existence of helices in the solid state and, hence, the contribute of helices to the total optical activity (from which it is also possible to infer if a preferential helix handedness exist) can be proved by evidencing optical activity or circular birefringence in laser conoscopic method. The optical phenomenon of circular birefringence or optical activity results in a rotation of the plane of polarization of linearly polarized light propagating in a medium, owing to the difference between the indices of refraction for the left and right circularly polarized components into which linearly polarized wave can be resolved. Evidencing circular birefringence by measuring the difference between refraction indexes for left and right circular polarized light is a way to assess optical activity and, consequently, the presence of chiral helical structures or any other type of enantiomerism in crystals. In other words, circular birefringence and circular dichroism exhibited by chiral substances afford a quantitative means for species discrimination. Unfortunately, circular dichroism does not apply to transparent materials and, moreover, the very low circular birefringence of solid polymer compared to linear birefringence makes measurements labor intensive with traditional procedures. Quantitative and qualitative measures of the chiroptical properties and the direction of rotation of the polarization plane for polymers can be easily obtained by means of laser conoscopic observations with the use of a λ/4 plate of known optical sign to produce spiral isochrome patterns [22,23].

As said before, a right-handed helix of a flexible chain could be turned into a left-handed helix, and vice versa, by completely unwinding and re-coiling in the opposite handedness. The interchange of helices with opposite handedness is, however, impossible in the solid phase, where allowed adjustments are too small to produce all conformational changes needed for this conversion. Therefore, if chiral helices developing during crystallization of a polymer cannot be included in the same crystalline lattice, as for instance happens for POM, they have to be enclosed in different crystals since the start of the solidification [7], thus originating a mixture. Although the crystal structure of macromolecules is apparently more complex than that of elemental and low-molecular-weight substances because of the high number of atoms into cells, the definition of polymer polymorphs does not differ, ultimately, from that of polymorphs of other compounds. Additional complexity is due to the possibility, for a same macromolecule, to show polymorphism in the solid phase not only because of different packing of a same chain conformation but also because of the possibility of different conformational isomers packed in different unit cells, as shown for POM. Conformational isomers differ for rotational angles around single bonds and, hence, form different helices which can be equivalent or not. Syndiotactic polypropylene, for instance, can crystallize according to two orthorhombic forms: one containing two equivalent enantiomorphic helices with opposite handedness and the other enclosing only chains in the planar trans-conformation, which can be considered as a degenerated form of a helix [24]. It is worth specifying that, differently from enantiomorphs, polymorphs show different thermal, and in general physical, properties.

### 2.2. Classical Nucleation Theory (CNT)

The classical nucleation theory was first developed by Volmer and Weber [25], Becker and Doring [26], and Zeldovich [27] to study first-order phase transitions as vapor condensation in liquid droplets and crystallization from melt. This theory divides the transitions in two distinct stages. In the first stage, particles of the new stable phase are formed because of random fluctuations of molecules in the metastable initial phase. The casual association of a small number of molecules is referred to as “nucleation.” In the subsequent stage, nuclei that have achieved a critical minimum size can grow regularly at expense of the initial phase, whereas particles smaller than critical nuclei will disaggregate. The formation of a new phase is favored by the higher thermodynamic stability due to a free energy gain, but it is hindered by the free energy cost associated to the formation of a surface area [28]. A high number of nuclei entails a high value of the area of the solid–liquid interface. The formation of an interface needs a work to be done as it increases the free energy of the system. Nuclei with a high unfavorable ratio between surface area and volume will be continuously formed and dissolved, whereas only nuclei able to reach a relatively considerable size will grow. In other words, very small nuclei of the more stable phase are thermodynamically less stable than the initial phase; therefore, in order to have the phase transition, the size of the nuclei must exceed a minimum. The free energy variation Δ*G* associated to the creation of a spherical particle with radius r is given by the sum of two terms:

(1) $$\Delta G=4\pi r^{2}\gamma +\frac{4}{3}\pi r^{3}\Delta G_{v}.$$

The first term of the Equation (1) is connected to the energy per surface unit γ, and the second term is connected to the energy change per volume unit $\Delta G_{v}$.

**Figure 4:**
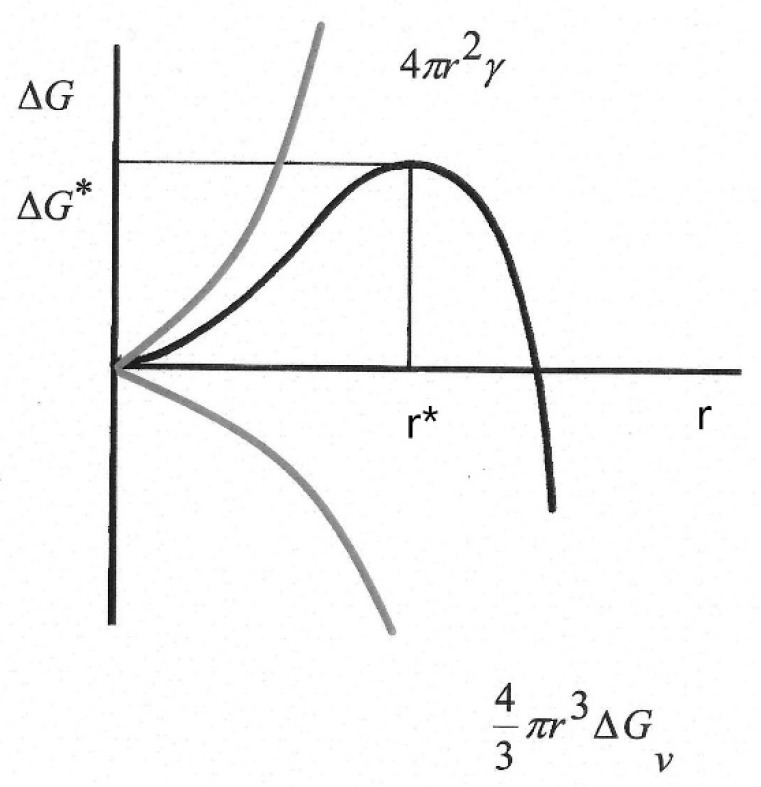
Free energy variation for nucleation (black curve) and its component functions (gray) expressed in Equation (1). After references [25,26,27].

As shown in Figure 4, the function ΔG increases with r up to a critical value $r^{*}$ and then decreases. Therefore, particles with size less than $r^{*}$ tend to dissolve as their formation have an unfavorable ΔG. Vice versa, particles with radius higher than $r^{*}$ tend to grow.

The value of the critical radius can be obtained observing that in correspondence to $r^{*}$, the free energy change ΔG is maximum and therefore its derivative must be zero:

(2) $$\frac{\partial (\Delta G)}{\partial r}=8\pi r\gamma +4\pi r^{2}\Delta G_{v}=r(8\pi \gamma +4\pi r\Delta G_{v})=0.$$

From this condition, it follows:

(3) $$r^{*}=\frac{-2\gamma }{\Delta G_{v}}.$$

The free energy change of critical particles is:

(4) $$\Delta G^{*}=\frac{16\pi \gamma^{3}}{3\Delta G_{v}^{3}}.$$

These critical parameters can be expressed as function of temperature by observing that at the fusion temperature, there must be solid–liquid equilibrium and therefore, $\Delta G_{v}=\Delta H_{v}-T_{f}\Delta S_{v}=0$. Ignoring the variation of the enthalpy and entropy with temperature, the free energy can be expressed as follows:

(5) $$\Delta G_{v}(T)=\Delta H_{v}-T\Delta S_{v}\approx \Delta H_{v}(1-\frac{T}{T_{f}})=-\Delta H_{v}\cdot \frac{\Delta T}{T_{f}},$$

where $\Delta T=T_{f}-T$. Thus, $r^{*}$ and $\Delta G^{*}$ can be rewritten as:

(6) $$r^{*}=\frac{2\gamma T_{f}}{\Delta H_{v}\cdot \Delta T}$$

(7) $$\Delta G^{*}=\frac{16\pi \gamma^{3}T_{f}^{2}}{3{\left(\Delta H_{v}\Delta T\right)}^{2}}.$$

The decrease in $\Delta G^{*}$ with the overcooling entails that at lower temperatures, the probability of an embryo to become a nucleus increases. This enhancement of probability reflects the higher nucleation density of spherulites at low crystallization temperatures. The CNT has been developed for homogeneous nucleation; the mathematical treatment for nucleation on foreign surface is more complicate as it must take into account the interfacial energies crystal-substrate $\gamma_{c}$, melt-substrate $\gamma_{m}$, and melt-crystal γ. Briefly, a low value of the difference Δγ:

(8) $$\Delta \gamma =\gamma +\gamma_{c}-\gamma_{m}=\gamma -(\gamma_{m}-\gamma_{c})$$

due to a high value of the difference $\gamma_{m}-\gamma_{c}$, indicates a more favorable nucleation process as the formation of an interface between the crystal and the substrate needs a lower energy cost.

Nevertheless, from the mathematical complications, it can be easily recognized that the effect of the temperature on the nucleation density is unchanged for heterogeneous nucleation.

The CNT assumes that the nuclei have a spherical shape due to the surface tension (i.e., the surface free energy per unit area) [29]. As all theories, the CNT is based on approximations that simplify the mathematical treatment. The most controversial assumption in CNT is the use of the macroscopic surface tension for the thermodynamic treatment of very small particles of the new phase [30]. A new nucleation theory [30,31,32], developed for liquid–solid and vapor–liquid transitions, is based on the motion of the single molecule and molecular interactions instead of macroscopic thermodynamics, thus overcoming the assumption of high surface energy of crystalline nuclei. The numerical results of this new theory are more accurate but consistent with the results of the CNT, therefore, only the nucleation theory has been briefly reviewed in this work.

### 2.3. Growth of Crystals: Thermomechanical and Secondary Nucleation Theories

Over the last 70 years, there has been remarkable progress in understanding of the growth process of crystals [33]. First-order phase transitions are characterized by two thermal processes, i.e., exchange of latent heat of phase transition and heat diffusion. In 1950s, the model of solidification was based on solute or thermal diffusion [33] and treated with the Stefan problem [34] under the assumptions of sharp solid–liquid interface (i.e., a zero-thickness interface) and isotropy. The classical Stefan model is a free boundary problem that represents thermal processes in phase transitions by accounting for heat diffusion and exchange of latent heat. Since heat conduction in a system where a phase transition occurs is a non-linear phenomenon, exact solutions to Stefan problem are obtainable under oversimplified boundary conditions, ignoring non-thermal processes as stress and deformation in the solid, convection in the liquid, change of density, surface tension, etc. The Stefan equations relay on the old observation of Gibbs that exothermal phase transitions need heat removal from the new phase in order to proceed and have been settled on the basis of the Fourier thermal conduction laws. Ivantsov [35,36] found analytical solutions to the Stefan problem for particular boundary conditions. He, ignoring the Gibbs-Thomson correction for capillarity in the special case of very rapid interface kinetics, considered the problems for which both the solid–liquid interface and the whole solid are at constant temperature, the thermodynamic melting temperature. Under these conditions, a family of surfaces known as quadric (ellipsoids, hyperboloids, and paraboloids and their degenerations such as spheres, cylinders, and planes) is obtained [33]. The evolution of these forms entails changes of size, but not of shape, i.e., the isotherms are members of the same class of quadric. Moreover, any linear dimension of these quadric surfaces increases in proportion to $t^{1/2}$. As the heat equation is parabolic [37], equations describing the solidification are not invariant under the transformation of t with −t, i.e., the behavior of a system is inherently different during crystallization and melting [33]. Ham [38] showed that some of these interfaces can be made to reverse by melting, but only for a finite time; Ham also found equations to determine growth and melting rates as function of overcooling or superheating, respectively. These are the only exact analytical solutions to the solidification problem with an isothermal interface [33].

But in 1960s, these solutions, on the basis of the experimentally observed cellular and dendritic shapes, were tested for morphological stability and shown to be unstable [33]. The theory of morphological stability described the stability of the shape of a solid–liquid interface during a crystallization process. Analysis of stability consists in introducing a small arbitrary perturbation in the shape of the interface and solves the problem for the perturbed interface in order to establish if the perturbation will grow (i.e., the original surface is unstable) or decay (i.e., the surface is stable) [33,39]. Mathematically, the morphological instability was studied by considering the growth of a sphere perturbed by spherical harmonic [33,40]. If the interfaces were isotherms near the perturbation, the interfaces would assume the same shape of the perturbation, enhancing the growth of the perturbation. However, non-isothermal interfaces reduce the shape distortion favoring stabilization. Detailed analysis leads to the conclusion that high overcooling of the melt, entailing a highly negative temperature gradient at the solid–liquid interface is destabilizing whereas the capillarity phenomenon is stabilizing.

Instability is generally observed near defects, such as grain boundaries. For instance, a grain boundary oriented perpendicularly to the solid–liquid interface generates a groove in the otherwise almost planar interface [41]. To deal with this instability, it is necessary to solve the Laplace’s equation describing the temperature field in the solid and in the liquid, taking into account the Gibbs-Thomson effect. This latter assumes the temperature of the solid–liquid interface as the melting temperature corrected for the local curvature of the interface and for the capillarity. It is thus possible to obtain three equations describing the shape of the interface as a function of time according to the values (negative, zero, and positive) of the sum of the temperature gradients, in the liquid and solid sufficiently far from the interface.

The theory of morphological stability is very general and applies to both growths by solution and by melt. It is worth noting that the presence of a grain boundary groove does not change the criterion for morphological instability, which is the exponential growth of perturbations, whereas stability entails the decay of perturbations [39]. For t→∞, a stable interface, whenever a grain boundary groove is present or not, achieves a time independent shape; instead, an unstable interface becomes sinusoidal with exponentially increasing amplitude. In real observations, it is difficult to establish the stability or instability of the interface from its shape, as oscillations may appear also in stable interfaces at not infinitive time. Exact solutions other than the shape preserving solutions considered above may be obtained under the limit condition that heat flow is so fast that the entire system (liquid, solid, and sharp interface) is at a uniform temperature. If the capillarity correction can be left out and the growth is slow enough, so that the temperature difference needed to remove the latent heat is negligible, the heat equations can be ignored. With these assumptions, the interface overcooling Δ*T* is constant and the growth rate of the crystal does not depend on time, but depends only, through a kinetic coefficient, on the crystallographic orientation. In this case, the shape analysis can be carried out in analogy to the so called Wulff construction of the equilibrium shape of a crystal, since the mathematic equations describing the two growth processes are similar.

The last approach to crystal growth and shape considers indispensable to include the capillarity effects in the computations and also account for the anisotropy of the surface tension that, in turn, originates anisotropic interface kinetics. These effects can be advantageously handled by replacing a sharp interface with a diffuse interface (phase field model) [33]. The phase field model requires a not immediate mathematical procedure even in the simple case of solidification of a pure material from melt in the approximations of isotropy of all quantities and absence of convective flows. However, its application gives differential equations for the knowledge of time dependence of temperature, composition and phase field. Moreover, it has led to a better understanding of the pattern of cellular and dendritic interfaces.

A theory that predicts the dependence of the growth rate on temperature is based on the secondary nucleation, consisting in the formation of secondary nuclei on a flat surface [42,43]. These nuclei generate steps on the surface that “move” with constant rate as new polymer stems add to the substrate at sites adjacent to the nuclei. These steps develop until they collide and then their growth on the substrate stops. Single crystals grown under control of nucleation result in well-defined habits with facets that are determined by the slowest growing planes [44]. Indeed, the shape of single crystals depends on the relative growth rates of their faces; high index faces grow fast and disappear, whereas slow growing faces determine the overall growth rate and shape of crystals [44].

## 3. Crystallization and Microstructure of Polycrystalline Materials

As the crystalline structure of ideal solids is assumed as a perfect three-dimensional repetition of a unit cell, it should not show discontinuities. In real solid, however, defects are unavoidable and the orientation of the crystalline lattice may vary by passing from a portion of the solid to another. These portions in metals are referred to as grains and may be recognized according to the orientation of the crystalline structure. By passing from a grain to another the lattice remains the same but the crystallographic directions change their spatial orientation; as a consequence, the interfaces between grains constitute discontinuity lines and affect the chemico-physical properties of the structure. The presence of such discontinuities is due to the initiation of the liquid–solid transformation in more than one point of the liquid [45]. Once formed, solid embryos grow by addition of fresh material to their surface [46]. In absence of solvents, polymer nuclei grow with a rounded shape because of the maximization, for bi-dimensional nuclei, of the surface area and a minimization of the interfacial energy [47]. Indeed, circular crystalline units known as spherulites are common in nature [48,49] and, in particular, are typical of polymers solidified from melt. Because of their spherulitic structure, polymers are considered polycrystalline materials and the growth of their basic crystalline units, i.e., spherulites, shows several analogies to that of grains in metals. The grains either begin to grow as soon as some critical undercooling is surpassed locally or are nucleated at a temperature dependent rate. The grains are assumed to be isothermal and growing at the local bulk undercooling [50]. A temperature difference will exist between a growing grain and the surrounding liquid owing to the latent heat being liberated as it grows, but this will be very small because the thermal profiles of neighboring grains will overlap owing to the high thermal diffusivity of metals [50]. However, this is not the case for polymers that have much lower thermal conductivity than metals.

The microstructure of materials is determined by the evolution of the domains occupied by the developing solid phase. The shape of the interface between two adjacent spherulites is also a useful source of information on crystallization. Interfaces between adjacent spherulites may be lines or curves according to both their relative sizes and linear growth rates [1]. Furthermore, the exact shape of interfaces depends on the ratio between the true or overall growth rates of the two coalescing spherulites. Only spherulites with equal life-times and linear growth rates, i.e., with the same true growth rate, show linear interfaces. Spherulites with the same linear growth rate, but different nucleation times, as well as spherulites with different linear growth rates and equal life-times, are separated by curved interfaces [1]. A predictive theory of the shape of spherulitic interfaces (which are usually conic sections, such as branches of hyperbola, parabolas, ellipses, and their degenerations), based on simply geometrical considerations, can be found in reference [1]. 

**Figure 5:**
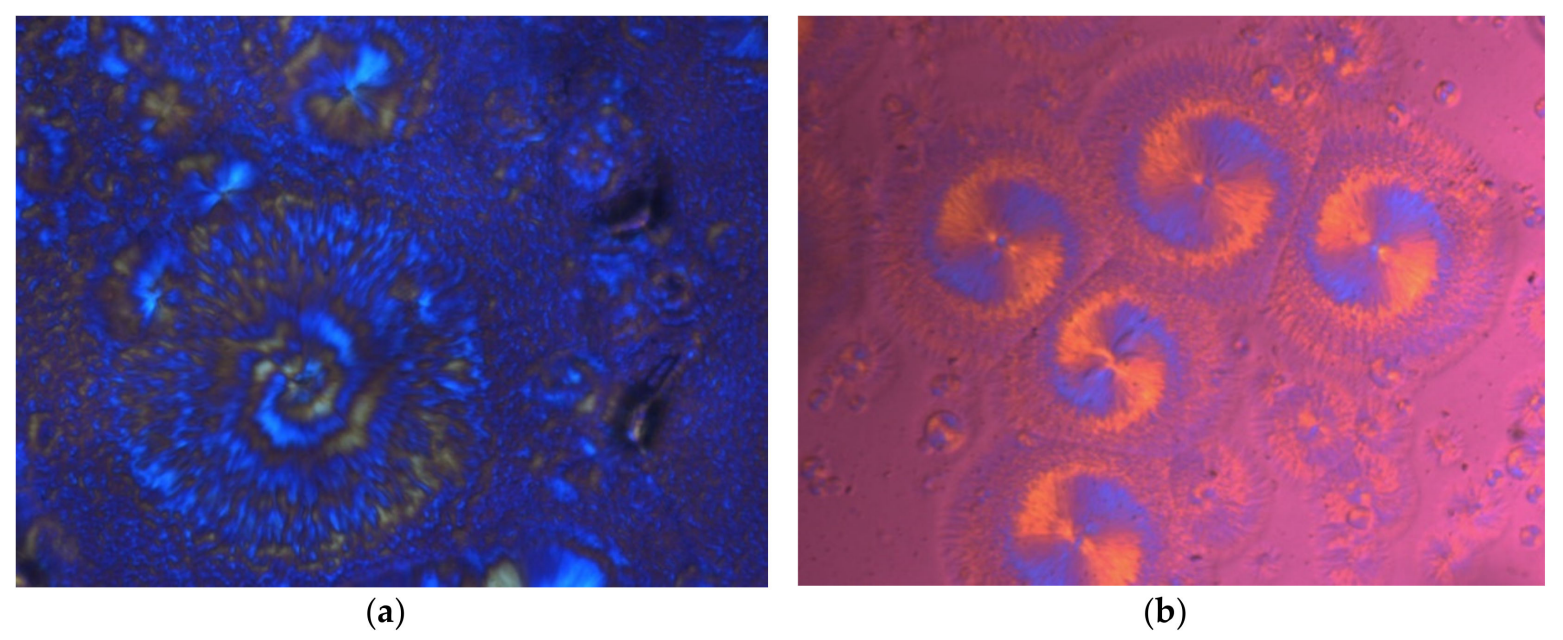
(**a**) Micrograph of spiral spherulites of PHB obtained with a blue filter and (**b**) colored micrograph of PHB spherulites taken in the presence of a lamina λ.

Most of spherulites grow by one of two mechanisms: extension and branching of centrally connected crystal rods or spiral winding of fibrils. Although radially grown spherulites are most commonly encountered, spirally grown spherulites have been observed by many authors [51,52,53,54,55,56]. Examples of spiral PHB spherulites are shown in Figure 5.

Often, spirally grown spherulites have shape and appearance hardly distinguishable from that of radial spherulites and form interfaces with exactly the same geometry of those formed by radial spherulites. However, independently of the growth mechanism, the external shape of spherulites may, sometime, deviate from a perfect circle for different reasons [57]. The exact geometry of the contour is almost as important as the inner structure since, together to the linear growth rates and nucleation times, it affects the shape of the interspherulitic boundaries and thus the mechanical resistance of materials [1].

Superstructures others than spherulites, called hedrites, are sometimes observed during crystallization of polymers from the melt. Hedrites have been defined as crystal aggregates with a complexity intermediate between single crystals and spherulites [57]. They consist of more or less parallel, centrally connected lamellar crystals with a polygonal appearance, such as squares and hexagons, when viewed flat on [58]. When hedrites are viewed from the edges of the lamellar stack, depending on the level of maturity, they show a lathlike, round, or oval shape [59]. Hedrites are formed occasionally and are not as representative of polymer solids as spherulites; hereinafter, hence, we will not deal with them.

## 4. Effect of Thermal Properties of Polymer Composites on Crystallization and Melting

As already discussed, crystallization is considered to occur in two steps, i.e., primary and secondary nucleation (i.e., growth). Secondary nucleation theory, based on kinetic parameters as the growth rates of crystal faces, is very useful to explain the shape, the interface stability of single crystals and dislocations in crystals. The morphology of polymers, however, is mainly governed by primary nucleation. The growth in the interior of metals of non-dendritic circular cells (i.e., grains), equivalent to polymer spherulites, is referred to as equiaxed. As non-dendritic equiaxed structure, spherulitic structures observed in polycrystalline substances are strongly affected by primary nucleation. Secondary nucleation, although important for the growth rate of all minute crystals forming spherulites and, hence, also for the size achievable by an ideal isolate spherulite, has a marginal role in the definition of the shape and the ultimate size of spherulites within polycrystalline sample. Indeed, the isotropic growth of thin radial crystals from a common center, entails that the growing fronts of all crystals form concentric circumferences and, hence, moving interfaces without faces. If dendritic growth occurs, it concerns the interior of spherulites at submicrometer size and does not affect the border and the shape of spherulites. Moreover, the size of spherulites depends on the distance amongst nucleation sites. In other words, the circular shape of polymer spherulites is determined by the radial arrangement or spiral winding of crystals, whereas secondary nucleation on the growth fronts of crystals has not relevance on the whole shape and the average dimension of spherulites, which is mainly determined by the nucleation density. The primary nucleation is even more important for polymer matrices in composites, where foreign particles act as discontinuity points of matrix properties, especially relatively to heat propagation. An underestimated and not yet deeply investigated phenomenon in polymer composites is thermal rectification, a property potentially exploitable in electronics and several other technologies [60]. Thermal rectification occurs when structures transfer heat asymmetrically, so that the heat flow capacity along an axis is higher in the forward direction than in the backward direction under the same absolute, but opposite in sign, temperature difference. In other words, such asymmetric structures behave as conductors in one direction and as insulators in the opposite direction when the temperature difference is reversed. Thermal rectification has been observed not only in composites and heterogeneous structures but also in bulk materials [61]. The effects are, however, often small and implementation is difficult [62]. In composites, the poor rectification can be ascribed to the thermal resistance at the interfaces. For instance, the calculated resistance at the interface between octane and single-layer graphene sheets (GS, which shows extremely high values of thermal conductivity, i.e., 3000–5000 Wm^−1^ k^−1^ [63]), has been reported to be 10 times smaller when the alkane chains are covalently bonded at the edges of these GSs [64]. Pal and Puri found higher rectification using a single-wall carbon nanotube (SWCNT) covalently bonded near one end to polyacetylene (PA) chains [65]. This composite structure shows rectification up to 204%, which is higher than the values reported for single wall carbon nanotubes (SWCNTs). Go and Sen have discussed the necessary conditions for rectification to occur in composites and bulk materials [61]. They also showed that if the thermal conductivity is separable with respect to the two independent variables, i.e., space and temperature, there is no rectification. However, separability is a sufficient condition: if there is no rectification, the thermal conductivity may not be separable. As a consequence, a necessary but not sufficient condition for thermal rectification is that the thermal conductivity of the material be non-separable. When two or more homogeneous materials are joined to form a composite wall, the thermal conductivity of the composite structure is potentially non-separable even if the thermal conductivities of the individual materials are, and, hence, rectification is possible. A composite structure shows discontinuities in properties and in the temperature profile, and these can affect the overall thermal conductivity [61]. If the thermal conductivities of both components have the same temperature dependence, though different spatial dependencies, the thermal conductivity function is separable and no rectification can occur. If instead the thermal conductivities of both materials have the same spatial dependence, but different temperature dependencies, rectification may occur. It may be possible to create a material with non-separable thermal conductivity, such as an alloy with a non-uniform distribution of one material in the other or with two or more dissimilar homogeneous materials where at least one of the thermal conductivities is a function of temperature [61]. Indeed, a way to originate a material potentially owning rectification properties consists in the use of two or more homogeneous substances whose thermal conductivities show different temperature dependency. As a matter of fact, the opposite temperature dependence of thermal conductivity for polydimethylsiloxane (PDMS) and graphite powder has been considered to be responsible for thermal rectification of their composites [65].

**Figure 6:**
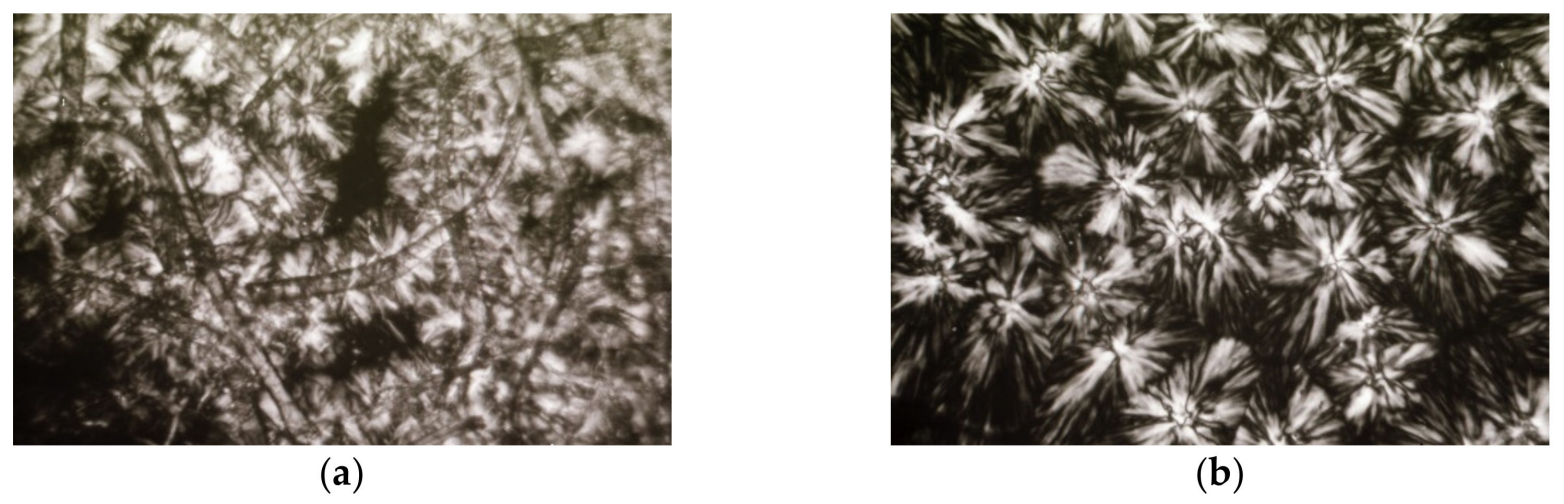
(**a**) Optical micrographs of short Kevlar fibers/isotactic polypropylene (iPP) crystallized by cooling from melt at −3 °C/min and (**b**) neat iPP crystallized under the same cooling rate, showing a coarser spherulitic structure.

In absence of relatively fast and continuous undercooling from melt, temperature fluctuations in polymers are not so wide for nucleation to occur, and consequently, crystallization is hindered or very slow. To speed up quiescent crystallization, high fluctuations have to be induced by continuous and deep undercooling. To explain the crystallization behavior and the microstructure of polymer composites, the model of solidification adopted for polycrystalline substances needs to be adapted to account for large differences between thermal properties of polymers and fillers in composites. Indeed, the presence of particles or fibers with thermal conductivity much higher than polymers and, usually, lower thermal capacities, affects considerably the solidification of composites. For instance, carbon nanotubes (CNT) even at low concentration can enhance the thermal conductivity of a polymer matrix at least of an order of magnitude [64]. As a result, composites usually show a finer spherulitic structure than the corresponding matrices, especially at high undercooling [66]. For instance, in Figure 6, samples of neat iPP and of a composite polyaramide (Kevlar) fibers/iPP crystallized under the same conditions are compared.

**Figure 7:**
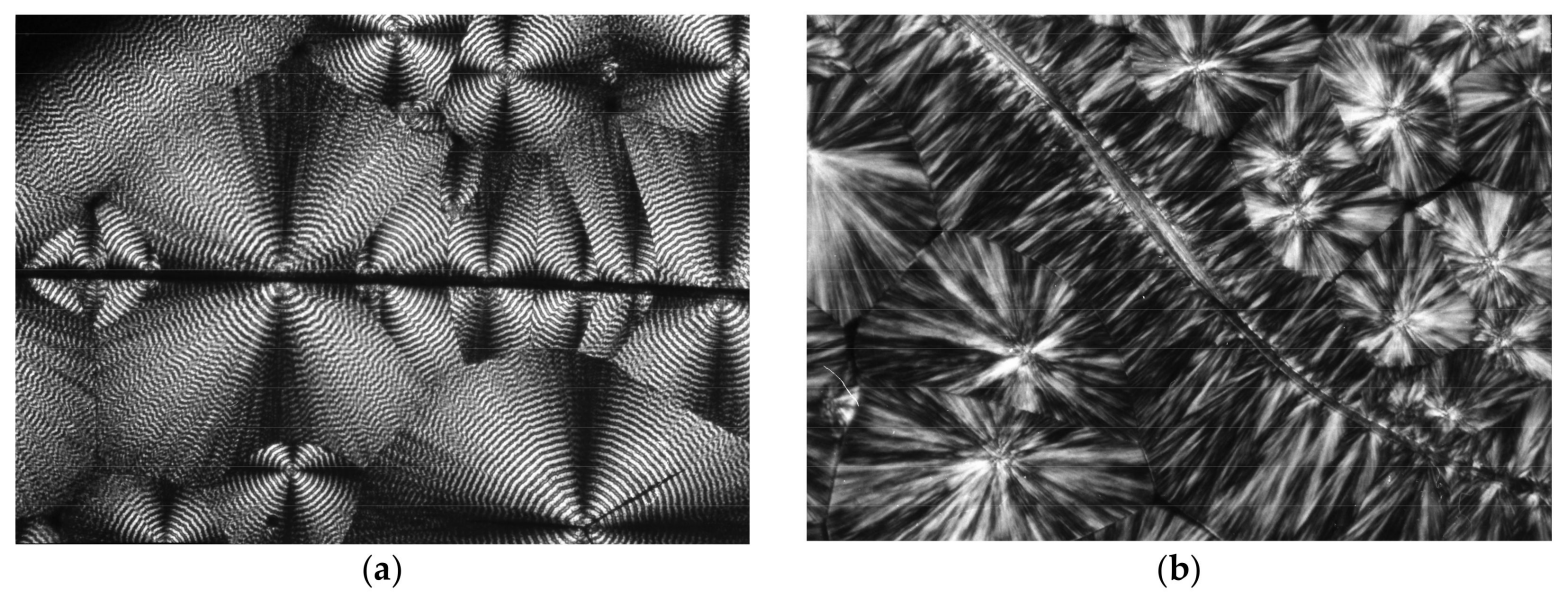
(**a**) Micrograph of columnar growth in PHB/Kevlar and (**b**) transcrystallinity in iPP/Kevlar.

Particulate and well-dispersed fillers generate a more or less uniform increase of nucleation in a thermoplastic matrix. Although each particle may, in principle, reach lower temperatures before the polymer matrix, acting, hence, as a nucleating site, in practice, this circumstance does not explain the high nucleation density in sample with a very low content of the filler, indicating that the whole increase of thermal conductivity speeds up local nucleation. Indeed, even in extremely pure substances, high undercooling produces increased nucleation density. Filler with anisotropic shape, as fibers, may induce also anisotropic effects, i.e., orientation, during nucleation and growth of semicrystalline polymers. The presence of foreign particles with rounded shape produces usually a denser nucleation of spherulites and, thus, a finer texture without any kind of orientation. Instead, fibers are inherently anisotropic with respect to heat propagation and generally show a longitudinal thermal conductivity much higher (even of a few orders of magnitude) than that of the same non-fibrous substance. Efficient heat removal needs a phonons flow through a continuous network, and well-dispersed particles, as well as short fibers, in the usual low content cannot form a conductive pathway. Fillers in any form and low amount, however, change the thermal properties of the polymer matrices and cause a reduction in the size of spherulites, with none or scarce other apparent morphological changes. Longer fibers emerging at the surfaces of composites, moreover, constituted preferential pathways through which crystallization heat can be transferred to the surrounding. Therefore, although nucleation in polymer composites generally entails a finer-grained structure than neat polymers, it may also lead to directional growth resulting in particular morphology along fibers, such as transcrystallinity (see Figure 7).

The phenomenon of transcrystallization may occur not only around foreign particles and fibers in polymer composites but also in neat polymers when nucleation originates from the coldest surfaces of the vessel. Transcrystallinity has been described by several authors and involves a wide range of surfaces such as aluminum, carbon, glass, as well as polymeric and natural fibers [67,68,69,70,71]. Almost all polymers show transcrystallinity layers around fibers, whose thickness and fineness are also related to the average size of bulk spherulites. For instance, as shown in Figure 7, iPP forms transcrystalline regions onto Kevlar fibers that are denser and thinner than PHB.

**Figure 8:**
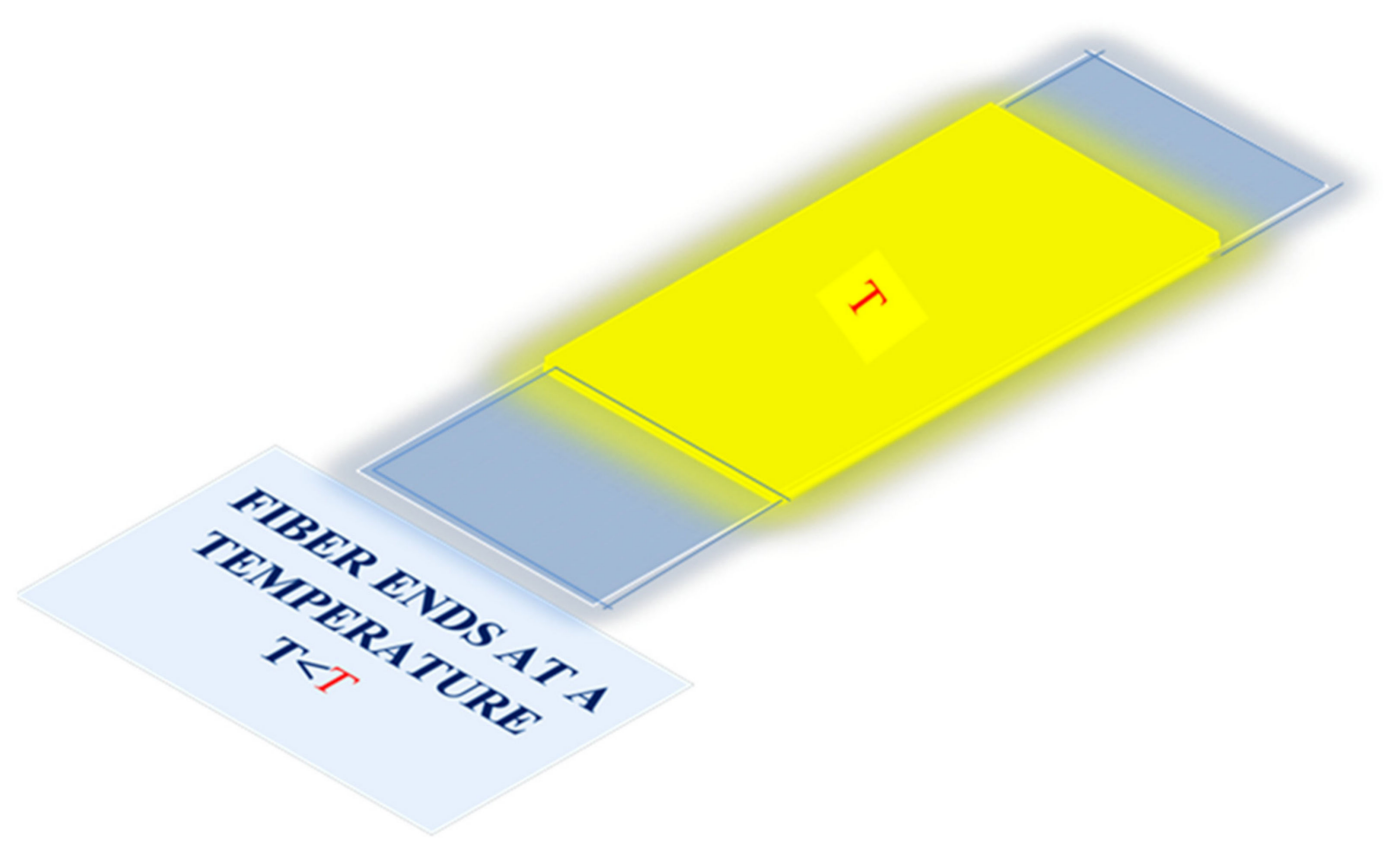
Possible use of fibers as heat sinks in composites.

The transcrystalline morphology is caused by the faster transport of the crystallization heat towards the external surfaces of the specimen through fibers. The higher the cooling rate and the longitudinal thermal conductivity of fibers, the closer the spherulites and the denser the transcrystalline layer. Despite the primary use of fiber composites as structural or reinforcing materials, embedding in a low thermal conductivity matrix several conductive fibers, leaving their ends in the surrounding, would be a useful way to avoid overheating of materials by efficient transport of heat from generating components to the surrounding, as shown in Figure 8.

**Figure 9:**
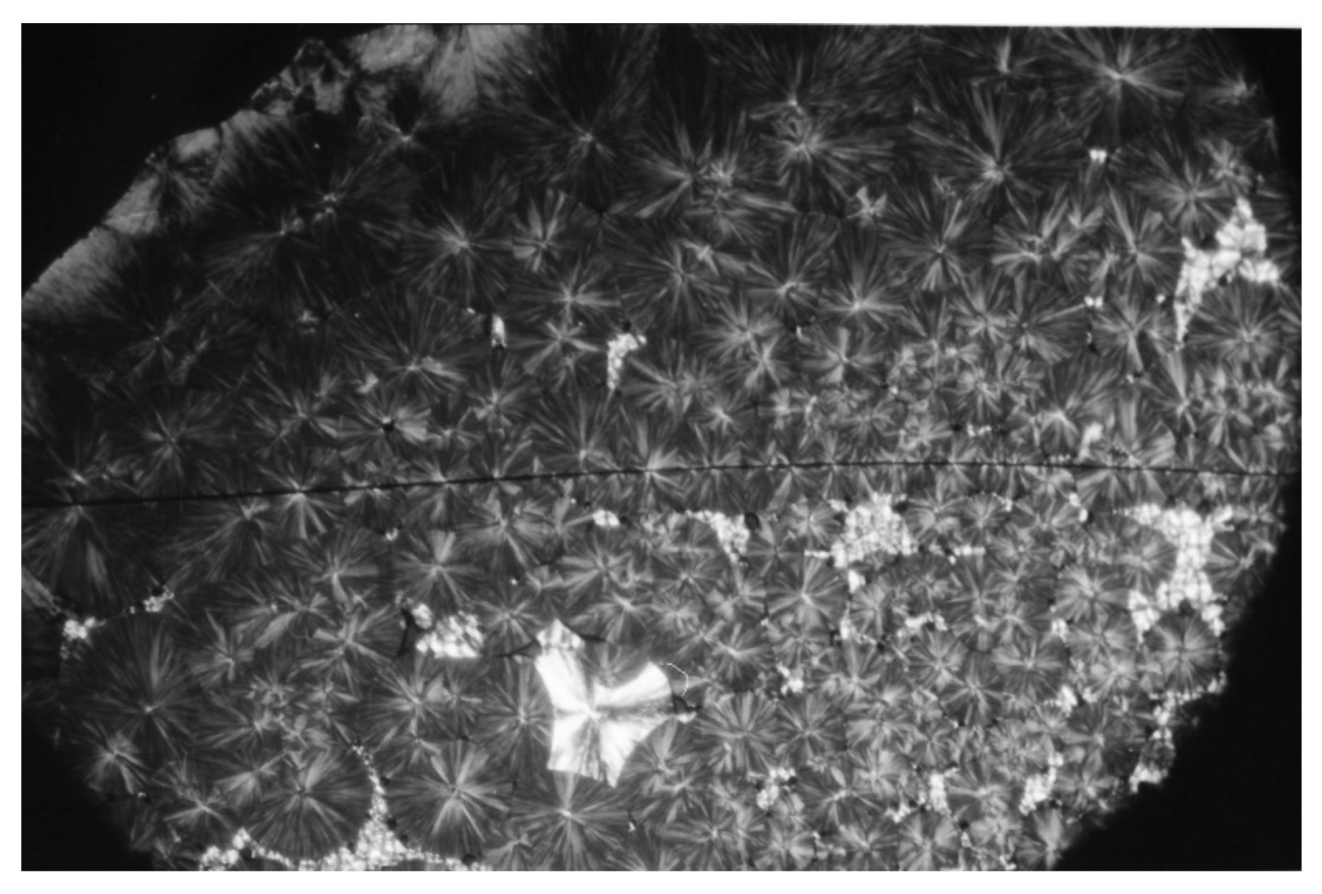
Differences in nucleation density of spherulites amongst regions within a specimen of iPP crossed by a carbon fiber.

For low conductive substances as polymers, a better agreement between theoretical and experimental crystallization behavior is achieved if nucleation is considered to be a deterministic, instead than a stochastic process [45,46,72,73,74]. Deterministic theories account for nonhomogeneous temperature fields and predict different spherulite densities in different parts of a sample [46], as shown that in Figure 9.

Deterministic models also assume the existence of a critical nucleation temperature above which the probability of nucleation is so low that it may not occur at all [72]. According to deterministic theories, if, at the solidification onset, steep temperature gradients exist at the borders of a sample, the temperature gradients result in crystallinity gradients, i.e., the growth front propagates from the edges towards the interior of the sample [72,75,76]. If nucleation were actually random in space, solidification of a specific substance would occur in a time independent of the mass and geometry of the sample. Actually, heat removal rate is not infinite [37], so that undercooling, and hence crystallization, advance from the external to the inner of the melt. This thermal effect may originate directional solidification with a columnar growth, a well-known phenomenon also occurring during small casting of metals and alloys both in single-phase and two-phase eutectic growth. Almost pure metals mostly freeze with a columnar structure [75]. During columnar growth, grains nucleated near the mold wall grow as a unique front towards the center of the casting. Under particular conditions, for instance, in larger casting, equiaxed growth can be promoted, and grains grow radially from many points of the casting. Columnar and equiaxed growth can also coexist. In metals, fully columnar growth is expected at low growth rates and high gradients, whereas the wideness of the equiaxed growth increases at high velocity and low temperature gradients. In polymer composites, the presence of foreign particles with thermal conductivity higher than that of the polymer matrix increases the nucleation density by increasing the rate of heat removal in the interior of the liquid matrix, thus accelerating the equiaxed growth of the solid phase. Since polymer composites cool faster than neat matrices, the undercooling needed for nucleation is achieved earlier. For instance, the crystallization time at 133 °C of iPP composites containing 10% by weight of carbon fiber (CF) is 1/5 of the time needed to completely crystallize the same amount of neat iPP [66]. The higher crystallization rate of a composite relatively to neat polymer matrix is readily appreciable by differential scanning calorimetry (DSC), since the crystallization peak appears after shorter cooling times during non-isothermal crystallization. Fibers originates also anisotropic effects on the spatial distribution of the nuclei and, consequently, on the geometry of the growth. Let us consider a thin film of a fiber/polymer composite, where coplanar bi-dimensional spherulites may arise randomly in bulk, whereas transcrystalline layers nucleate onto fibers; neglecting the short final stage of coalescence amongst spherulites, the overall crystallization rate depends on the growth rate of both bulk spherulites and transcrystalline layers and, hence, also on their specific nucleation times.

**Figure 10:**
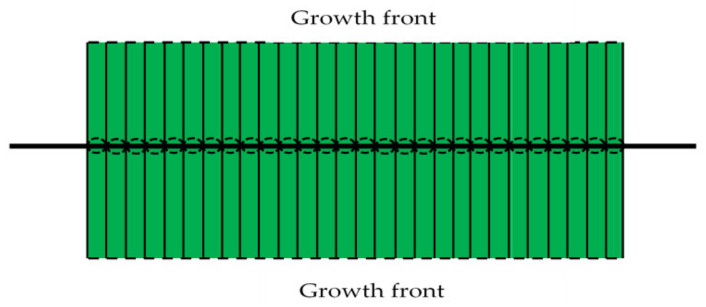
Diagram of growth of a dense transcrystalline layer around a fiber. Spherulites, initially rounded, after lateral coalescence adopt an almost rectangular shape and continue to grow, transversally to the fiber axis, at two opposite growth fronts.

Consequently, even if in fiber composites showing transcrystalline morphology the crystallization from melt starts early than in the neat polymer matrix, the growth rate increase during solidification may be lower than that occurring in the neat polymer matrices. See, for instance, the sketch of Figure 10, where nucleation along a fiber is so dense that the lengthening of the growth fronts of the arising transcrystalline layer is approximately constant and, as a consequence, the growth rate is also almost constant.

Indeed, assuming as zero times the respective onsets of crystallization, the same number of nuclei in a neat matrix and in a transcrystalline fiber composites will growth with different instantaneous rates because of the limited one-dimensional growth of transcrystalline layers. This effect can be appreciated by comparing the results of the Avrami analysis for neat matrices and composites. Knowing the crystallinity fraction X(t) developed at various times during crystallization, the logarithmic form of the Avrami equation:

(9) ln[−ln(1−X(t))]=lnk+clnt

allows to determine easily two useful parameters, referred to as kinetic constant k and Avrami exponent c. This latter depends on the geometry of the growth and, therefore, assuming instantaneous nucleation, c should be equal to 1 for a fully transcrystalline sample and 2 for a fully spherulitic samples. Since higher nucleation rates of spherulites, i.e., lower induction times in isothermal crystallization, do not affect the value of the exponent of the Avrami equation, an index c inferior to 2 indicates a mixed one- and two-dimensional growth geometry and conforms to the presence of transcrystallinity in composite films. For instance, poly(ether ketone ketone) (PEKK) crystallization isotherms produce Avrami lines with equal slopes: the Avrami index *c* is neither affected by temperature nor by the presence of carbon fibers [77]. The crystallization rate depends on k, which increases with the supercooling for both neat PEKK and PEKK/carbon fiber composites. Moreover, in this composite, carbon fibers have the same effect of a uniform temperature decrease on the PEKK crystallization. This means that the spherulitic morphology of the PEKK matrix is preserved in the composite and orientation along the fibers is not expected. On the contrary, poly(ether ether ketone) (PEEK) shows a columnar growth on carbon fibers that causes not only an increase of k but also a reduction in *c* because of the reduced dimensionality of transcrystalline growth [78]. Li et al. compared the c and $Z_{c}$ coefficients obtained by the Jeziorny-modified Avrami equation for non-isothermal crystallization kinetics of CFs/PEEK and polyimide-modified CFs/PEEK composites [79]. The authors found that in the presence of unmodified CFs, the PEEK matrix shows higher crystallization rate constants $Z_{c}$ and lower Avrami exponents c. These findings have been ascribed by the authors to the higher thermal conductivity difference between the neat CF reinforcement and PEEK which induces transcrystallinity in composites, whereas the reduced thermal conductivity of polyimide-modified CFs cannot enhance the nucleation along fibers producing a high growth dimensionality and higher crystallization times. For a deeper discussion. the present author refers to the quoted literature. Briefly, a faster crystallization in composites can be due to a higher nucleation density of spherulites in the bulk matrix or to a massive nucleation along fibers. However, a high number of spherulites per volume unit does not change the Avrami exponent c, whereas the presence of transcrystallinity causes also a reduction in c with respect to the neat matrix.

Linear growth rates of spherulites and transcrystalline layers in composites are usually not found different from those of the neat matrices. However, it is worth observing that a possible linear growth rate enhancement at the beginning of isothermal crystallization is not appreciable because of the low magnification and resolution of optical microscopes, whereas under non-isothermal conditions, experimental limits connected to the continuous increase of the growth rate make the measures unreliable.

The presence of fillers can also induce, under particular cooling conditions, the nucleation of polymorphic forms that are absent, or present in a negligible amount, in neat matrices. The increase of the nucleation rate of a certain polymorph may, in limited cases, be ascribed to epitaxy, i.e., oriented growth of separate crystals of a material onto another substrate material due to polymorphism or, at least, close spacing between the constituent atoms of the two overlapped materials [80]. For instance, epitaxial growth may occur when polymeric fibers are used as filler of polymer matrices. In general, however, the effect of a foreign solid substance on crystallization of a polymer melt can be explained by adsorption and chemico-physical interactions that reduce the energy barrier for nucleation of a particular polymorph or by the steepness of local temperature fluctuations in the melt favoring the formation of polymorphs thermodynamically stable at lower temperatures. For instance, the incorporation of modified MoS_2_ in polyvinylidene fluoride (PVDF) induces the formation of the piezoelectric β phase [81]. Several “β nucleating agents” have been reported for isotactic polypropylene, whose crystallization under usual conditions leads instead to the monoclinic α phase. The increase of the amount of the hexagonal β phase in polypropylene composites is likely due to the higher cooling rate caused by the fillers, since thermal gradients alone also increase the proportion of the β phase in neat polypropylene [82]. Similar effects have been reported for several polymers [83].

When highly conductive fillers, in appropriate amounts, produce a continuous network within the polymer matrix, the enhancement of nucleation is closely correlated to the inherent high conductivity of the fillers. Through the network, indeed, the crystallization heat can be easily transferred to the surfaces of the samples and, hence, to the surrounding. However, the nucleating effect can be remarkable even for very low contents of fillers since solid particles are more responsive to the cooling than the liquid polymer matrix, thus inducing early nucleation in the local surrounding area. The presence of additives in polymers may also affect the melting behavior. An accurate measure of the melting point detected at the disappearance of linear birefringence under an optical polarizing microscope requires heating at a very small rate (not higher than 1 °C/min or even less for low thermal conductive samples) in a sufficiently wide temperature range around the expected transition temperature. An empirical measure of the adhesion between fibers and polymer matrixes is based on the visual observation of the fusion of a specimen formed by a long fiber with a relatively high diameter embedded in a polymer and heated at relatively high rate in the central portion. If the ends of the fiber can exchange heat with the surrounding, supposed at much lower temperature, a delay of the melting of the polymer immediately around the fiber, relatively to the bulk, could be appreciable when the fiber/matrix adhesion is strong. When fibers are loosed from the matrix, the fusion of a polymer around a fiber is observed to occur simultaneously to the whole bulk. This technique is applicable when it has been assessed by appropriate investigations that only one crystal modification of the polymer occurs in the whole composite. In some cases, indeed, fillers may induce the crystallization of polymorphs, which usually do not form in the neat matrix crystallized under the same conditions, causing the appearance of multiple melting. Miao et al. prepared composites of high-density polyethylene (HDPE) with ultra-high molecular weight polyethylene (UHMWPE) fibers by solution crystallization [84]. They ascribed the high temperature of double melting peak of the composites to the transcrystalline HDPE grown close to the surface of fibers and the low temperature peak to the fusion of the bulk HDPE. Multiple melting, however, may originate for different reasons, as, for instance, the production of low-molecular-weight species by chain scission, isomerization and, in case of polyesters, transesterification reactions during preparation and processing of polymer composites.

## 5. Literature Examples of Microstructure and Morphology of Thermoplastic Composites

The main characteristic of thermoplastic composites is a finer spherulitic structure in comparison to neat matrices. Basturk et al. [85] prepared composites of strontium titanate (SrTiO_3_) nanoparticles (600–800 nm average particle size) with poly(butylene terephthalate) (PBT) and linear low-density polyethylene (LLDPE). SrTiO_3_ nanoparticles tended to be agglomerated from 600 nm up to a few microns in size; an increase in the size of the agglomerates, with increasing the SrTiO_3_ content, was found. Nevertheless, the authors estimated a homogenous dispersion of the particles in the polymer matrices up to loadings of 50 vol%. Moreover, they found that SrTiO_3_ increases the crystallization temperature and reduces the crystallinity of both polymers. The authors also found a DSC double melting peak for PBT. They were not interested in investigating the nature of the double peak, but it is likely that the higher melting temperature β form of PBT occurs together to the α form because of the stress during composites preparation and processing [86]. The high onset crystallization temperature of the matrix in particulate and short fiber composites is a universal behavior found, with a very few exceptions, in almost all materials. The reason of the earlier crystallization from melt of most of polymer composites is the higher thermal conductivity of the heterogeneous material relatively to the neat matrix. Excluding the case of the faster nucleation of unusual polymorphs at higher local undercooling originated by the filler, a few exceptions to earlier crystallization regard some composites with unidirectional long fibers. These latter may hinder the fast heat removal from the matrix during cooling when poor adhesion between fiber and matrix exists. Indeed, heat exchange between two components is controlled by contact resistances, which increase with increasing the amount of air trapped at the interface. For instance, Lee et al. obtained a highly thermal conductive composite by stacking a carbon fiber/aromatic polyamide unidirectional prepreg [87]. The authors investigated the thermal behavior of the long fiber composite by DSC and, since the adhesion between the components was only relatively good, the composite showed a delayed crystallization peak by continuous cooling from melt, notwithstanding the high conductivity of the prepreg. Sommereyns et al. studied the influence of a cover of sub-monolayer carbon nanoparticles of polyamide 12 (PA12) powder particles on the kinetics and thermodynamic of the polymer crystallization, by means of DSC and fast scanning calorimetry (FSC) [88]. As expected, it was found that lowering the cooling rate entails higher undercooling for crystallization of PA12 to start, whereas the conductive carbon nanoparticles on the surface of polymer particles caused a shift of the crystallization at lower undercooling, producing a faster crystallization and the deformation of spherulites in ellipsoidal crystalline structures. de Paula Cavalcante et al. [89] added hydroxyapatite (Hap) nanoparticles to an immiscible blend of poly(3-hydroxybutyrate) (90 wt%) and polycaprolactone (10 wt%). It was found that Hap is selectively localized in the PHB phase and strongly influenced the PHB crystallization behavior. DSC and optical microscopy results indicated that hydroxyapatite nanoparticles acted as a nucleating agent. However, no significant changes in crystallinity degree were observed by wide angle X-ray diffraction (WAXD). Fractionated crystallization was observed for PCL and the increase in the Hap concentration promoted heterogeneous nucleation on the PCL droplets surface due to the nanoparticles close to the interface. Li et al. [90] prepared fully biodegradable poly(ethylene succinate) (PES)/cellulose rod-like nanocrystals (CNCs) composites, finding that at contents below 0.5 wt%, CNCs act as an efficient nucleating agent for PES, i.e., increase the spherulites’ number, reduce the spherulites’ size as well as the crystallization induction time, the crystallization half-time, and overall crystallization time, although CNCs do not induce modification of the crystal form. The non-isothermal melt crystallization behavior of PES in its composites was investigated at a cooling rate of 5 °C/min. Neat PES displayed a crystallization temperature of 37.7 °C, which was significantly increased to 61–63 °C for PES/CNCs 0.25–1.0 wt%. It has also been reported that CNCs also act as an efficient nucleating agent at low loadings for the crystallization of different matrices, such as poly(l-lactic acid), poly(butylene adipate-co-terephthalate), poly(ε-caprolactone), poly(butylene succinate), poly(butylene succinate-co-butylene adipate), and poly(ethylene adipate) [90].

Luo et al. [91] obtained in situ microfibrillar composites (MFCs) with isotactic polypropylene as matrix and β-nucleating agents (β-NAs) filled polyamide 66 (β-PA66) as dispersed microfibrils through multistage stretching extrusion technology. Since interfacial interaction between microfibrils and matrix in MFCs plays a crucial role in obtaining high-performance materials, the authors aimed at originating a transcrystallinity layer of β-iPP around PA66 microfibrils due to the diffusion of β-NAs to the interface. As expected, the crystallization behavior of iPP was strongly affected by the presence of PA66 microfibrils (diameters in the range of 1–5 μm). Indeed, the onset of the DSC crystallization peak dramatically increases with the incorporation of PA66 microfibrils and the increase was even higher with the incorporation of β-PA66. Conformably, the isothermal crystallization peaks of the composites were narrow in comparison with neat iPP, indicating a faster crystallization. Briefly, the literature shows that generally polymer composites crystallize at a high velocity, exhibit a finer structure than the pure matrices with the occurrence of directional crystallization and may also melt at different temperatures either because of different heat transfer patterns owing to poor adhesion between components or to the formation of different polymorphs. The main characteristics of composites and the differences from the neat matrices are reported in Table 1.

## 6. Conclusions

Even if a universal cause of the spherical geometry of crystalline aggregates has not yet been identified, supersaturation and isotropy of the liquid medium with respect to heat conduction must play an important role in spherulites formation. The spherical geometry is likely correlated to the energy gain resulting from the aggregation of elongated crystals in circular superstructures that may grow with the minimum surface energy, dissipating the heat of solidification with the same rate in all spatial directions. Polymers and fillers have very different thermal conductivity, and this difference is responsible for finer microstructures and transcrystalline morphology found in composites. The presence of foreign particles and fibers affect both the final shape and the number of spherulites arising for surface unit. The enhancement of the nucleation density of polymer matrix reflects the high thermal conductivity and low heat capacity of isotropic fillers, whereas anisotropic fillers like fibers may cause also orientation and anisotropic growth of polymer spherulites, resulting in unusual morphology as transcrystallinity. Understanding the local heat exchange in composites is essential not only for the production of materials with tailored microstructures and properties, but also to offer new applications and technologies. Indeed, polymer composites might show thermal properties, such as rectification, of great potentiality for the use in unconventional fields for plastics.

## Figures and Tables

**Table 1:** Comparison between characteristics of neat polymers and composites.

	Morphology	Polymorphism	Microstructure	Thermal Effects
Neat polymer matrices	Mainly equiaxed morphology ^a^	One polymorph is normally found under specific solidification conditions ^c^	Coarse spherulitic structures ^d^	Possible but unusual ^f^
Composites	Mixed equiaxed-columnar morphology ^b^	Mixture of polymorphs or a polymorph different from that formed by the neat polymer ^c^	Finer spherulitic structure ^e^	Very likely ^f^

^a^ [1,45,46,47,48,49,50,57], ^b^ [67,71], ^c^ [81,83,92], ^d^ [93], ^e^ [66,93,94], ^f^ [60,61,62,63,64,65].
